# Environment characterization and genomic prediction for end‐use quality traits in soft white winter wheat

**DOI:** 10.1002/tpg2.20128

**Published:** 2021-08-15

**Authors:** Meriem Aoun, Arron Carter, Yvonne A. Thompson, Brian Ward, Craig F. Morris

**Affiliations:** ^1^ Dep. of Crop and Soil Sciences Washington State Univ. Pullman WA 99164 USA; ^2^ USDA‐ARS Western Wheat & Pulse Quality Laboratory Washington State Univ. Pullman WA 99164 USA; ^3^ USDA‐ARS Plant Science Research Campus Raleigh NC 27695 USA; ^4^ Dep. of Horticulture and Crop Science Ohio State University Wooster OH 44691 USA

## Abstract

End‐use quality phenotyping is laborious and expensive, thus, testing may not occur until later generations in wheat breeding programs. We investigated the pattern of genotype × environment (G × E) interaction for end‐use quality traits in soft white wheat (*Triticum aestivum* L.) and tested the effectiveness of implementing genomic selection to optimize breeding for these traits. We used a multi‐environment unbalanced dataset comprised of 672 breeding lines and cultivars adapted to the Pacific Northwest region of the United States, which were evaluated for 14 end‐use quality traits. Genetic correlations between environments based on factor analytic models showed low‐to‐moderate G × E interaction for most traits but high G × E interaction for grain and flour protein. A total of 40,518 single‐nucleotide polymorphism markers were used for genomic prediction. Genomic prediction accuracies were high for most traits thereby justifying the use of genomic selection to assist breeding for superior end‐use quality in soft white wheat. Excluding outlier environments based on genetic correlations between environments was more effective in increasing genomic prediction accuracies compared with that based on environment clustering analysis. For kernel size, kernel weight, milling score, ash, and flour swelling volume, excluding outlier environments increased prediction accuracies by 1–11%. However, for grain and flour protein, flour yield, and cookie diameter, excluding outlier environments did not improve genomic prediction performance.

## Core Ideas

- End‐use quality traits are laborious and expensive to phenotype.
- Factor analytic models were successful in understanding genotype × environment interaction.
- Genomic prediction accuracies were high for most end‐use quality traits in soft white wheat.
- Environment clustering and genetic correlations identified outlier environments.
- For some traits, excluding outlier environments improved prediction performance.

**Abbreviations**
AACC: American Association of Cereal Chemists
AIC: Akaike information criterion
BIC: Bayesian information criterion
BLUE: best linear unbiased estimator
BLUP: best linear unbiased prediction
DAV: Davenport
DAY: Dayton
FA: factor analytic model
FSV: flour swelling volume
G × E: genotype × environment interaction
GBLUP: genomic best linear unbiased prediction
GBS: genotyping‐by‐sequencing
GEBV: genomic estimated breeding value
MAF: minor allele frequency
MLM: mixed linear model
PULL: Pullman
RITZ: Ritzville
SDS sedimentation: flour sodium dodecyl sulfate sedimentation volume
SKCS: Single Kernel Characterization System
SNP: single‐nucleotide polymorphism
WALLA: Walla Walla
WATER: Waterville
Water SRC: water solvent retention capacity.

## 1 INTRODUCTION

Soft white wheat (*Triticum aestivum* L.) produced in the Northwestern region of the United States has some of the highest end‐use quality in the world (Sjoberg et al., 2021). To meet the requirements of grain markets, millers, and bakers, maintaining or improving end‐use quality is a major component of wheat breeding programs (Zhang et al., 2021). Grain hardness (kernel texture), flour water absorption, protein (gluten) strength, milling quality, and baking quality differentiate hard wheat and soft wheat. Compared with soft wheat flours, hard wheat flours have larger particle size, higher damaged starch, stronger gluten, higher levels of non‐starch polysaccharides, and higher water absorption capacity. Thus, hard wheat is generally used for making bread, whereas soft wheat is used for making cookies, cakes, pastries, crackers, and Asian‐style noodles (Morris & Rose, 1996; Souza et al., 2002).

Phenotyping end‐use quality traits is laborious and expensive and requires relatively large amounts of grain; therefore, extensive quality analyses are often not performed in breeding programs until advanced generations (Jernigan et al., 2018; Kiszonas et al., 2013). Effective approaches to optimize phenotyping and selection can result in more efficient breeding for end‐use quality. For instance, understanding genotype × environment (G × E) interaction can optimize the number of environments that breeders should consider in testing breeding lines for end‐use quality. In the case where a particular trait is associated with high G × E interaction, the breeder should consider testing in many environments, whereas in the case of low G × E interaction, the breeder can test in fewer environments, which saves time and cost and facilitates testing of more plant materials in the breeding program.

Understanding G × E interaction is one of the key challenges in plant breeding (Dias et al., 2018). Proper understanding of G × E interaction offers the opportunity to characterize mega‐environments, to target environments to maximize genetic gains, and to evaluate genotype stability across environments (Crossa, 2012; Dias et al., 2018; Malosetti et al., 2013). Multiple methods have been used to study G × E interaction including linear models and multivariate models such as additive main effects and multiplicative interaction (Gauch, 1988) and the genotype plus G × E models (Yan et al., 2000). However, these methods have their limitations to accommodate multi‐environment and unbalanced datasets (Dias et al., 2018; Sjoberg et al., 2021; Smith et al., 2001). Thus, multiplicative mixed models such as the factor analytic (FA) model have been proposed as a more robust method to improve the understanding of G × E interaction in unbalanced multi‐environment datasets that are common in applied breeding programs (Beeck et al., 2010; Burgueño et al., 2008, 2011; Kelly et al., 2007, 2009; Piepho, 1998). The FA model is a variable reduction method that fits many variables into a few unobserved variables or latent factors and showed its utility in capturing the variance structure of G × E in multi‐environment trials (Cullis et al., 2010; Dias et al., 2018; Oliveira et al., 2020; Sjoberg et al., 2021; Smith et al., 2015).

**Core Ideas**

- End‐use quality traits are laborious and expensive to phenotype.
- Factor analytic models were successful in understanding genotype × environment interaction.
- Genomic prediction accuracies were high for most end‐use quality traits in soft white wheat.
- Environment clustering and genetic correlations identified outlier environments.
- For some traits, excluding outlier environments improved prediction performance.

Since end‐use quality traits are predominately controlled by genetic factors (Carter et al., 2012; Smith et al., 2011; Souza et al., 2012), the use of molecular markers for marker‐assisted selection or genomic selection can optimize breeding for end‐use quality traits (Kiszonas & Morris, 2018; Sandhu et al., 2021). The use of molecular markers helps selection for end‐use quality traits in earlier generations along with agronomic traits and plant resistance to abiotic and biotic stresses (Jernigan et al., 2018). Genomic selection has been proposed to predict quantitative traits (Meuwissen et al., 2001) to select superior lines using genome‐wide marker data before estimating their actual performance. Advances in DNA sequencing technologies enabled cost‐effective use of genome‐wide single nucleotide polymorphism (SNP) markers in genomic selection (Poland & Rife, 2012; Poland et al., 2012b). Currently, the cost of genotyping‐by‐sequencing (GBS) of ∼10,000 lines is equivalent to phenotyping 2,000 lines for processing and end‐use quality (Battenfield et al., 2016). Previous studies demonstrated the effectiveness of genomic selection for end‐use quality traits (Battenfield et al., 2016; Hayes et al., 2017; Michel et al., 2018) in hard wheat (bread wheat). However, to our knowledge there were no investigations on genomic selection for end‐use quality traits in soft white wheat that has some different milling and baking parameters needed for making other food products such as cookies and cakes.

Previous studies implemented genomic prediction to characterize environments (Dawson et al., 2013; Heslot et al., 2013). These studies showed that genomic prediction enables environment clustering analysis in unbalanced historical data. As clustering analysis requires a complete distance matrix between environments, genomic prediction allows the prediction of genotype performance in each environment (Dawson et al., 2013). On the other hand, Heslot et al. (2013) used genomic prediction to characterize environments by estimating marker effects in each environment rather than genotype performance in each environment. Therefore, genomic prediction can overcome the limitation of unbalanced datasets and facilitates environment characterization.

In this study, we tested the use of FA models and environment clustering based on genomic prediction to understand the pattern of G × E interaction for end‐use quality traits. To test these approaches, we used historical multi‐environment data on 14 end‐used quality traits from the Washington State University (WSU) soft white winter wheat breeding program. Using these datasets, the objectives of this study were to (a) understand and compare the pattern of G × E interaction for 14 soft white wheat end‐use quality traits, (b) identify possible environment clustering or outlier environments, (c) test the utility of genomic selection for end‐use quality traits, and (d) test the effects of excluding outlier environments on genomic prediction performance.

## 2 MATERIALS AND METHODS

### 2.1 Plant materials

A total of 672 soft white winter wheat breeding lines and cultivars selected from F_4:5_ derived lines and double haploid lines were used in this study (Supplemental Table S1). This population was evaluated in 29 environments (year–location combinations). These genotypes were grown from 2015 to 2019 in seven locations in Washington State (WA), USA: Pullman (PULL, 46.7298° N, 117.1817° W), Lind (LIND; 46.9721° N, 118.6153° W), Davenport (DAV; 47.6540° N, 118.1500° W), Ritzville (RITZ; 47.1274° N, 118.3800° W), Waterville (WATER; 47.6471° N, 120.0712° W), Walla Walla (WALLA; 46.0646° N, 118.3430° W), and Dayton (DAY; 46.3238° N, 117.9724° W). The wheat genotypes were evaluated as part of the WSU winter wheat breeding program. Lines in preliminary and advanced yield trials were selected for agronomic and disease resistance traits, and only superior genotypes were tested for end‐use quality. In this dataset, there were 1–7 nurseries in each environment and a total of 76 nurseries. During selection and advancement of breeding lines in the program, a given genotype was tested for end‐use quality in at least one environment. Some genotypes (mainly cultivars) were tested for end‐use quality in multiple nurseries per environment (replication within environment), whereas most breeding lines were tested in a single nursery per environment. From each nursery, a single sample from one replicate per genotype was tested for end‐use quality traits. In all years, Lind and Pullman had the highest number of nurseries and genotypes (*n* = 55–166), whereas Ritzville and Waterville had one nursery per year and the lowest number of genotypes (*n* = 13–25) (Supplemental Tables S2, S3). This is because only superior breeding lines that were selected from Lind and Pullman were tested in the remaining five locations in WA. The dataset was unbalanced with only 43 genotypes out of the 672 grown in more than 25% of the environments.

### 2.2 Phenotyping

The wheat genotypes were tested for 14 end‐use quality traits including Single Kernel Characterization System (SKCS) hardness, SKCS size, SKCS weight, test weight, grain protein, break flour yield, flour yield, milling score, ash, flour protein, flour sodium dodecyl sulfate sedimentation volume (SDS sedimentation), water solvent retention capacity (water SRC), flour swelling volume (FSV), and cookie diameter (Supplemental Table S1). These traits are classified into four categories, which are grain characteristics, milling traits, flour parameters, and baking parameters as described in Table 1. In soft white wheat, high SKCS size, SKCS weight, test weight, break flour yield, flour yield, milling score, and cookie diameter, and low SKCS hardness, grain and flour protein, ash, and water SRC are preferred. Flour swelling volume and SDS sedimentation volume may be high or low depending on the end use.

**TABLE 1:** Summary statistics of the soft white wheat end‐use quality traits

Trait	Unit	No. of observations	No. of genotypes	Min.	Max.	Mean	SE^a^	*H* ^2^ ^b^
Grain characteristics								
SKCS hardness	unitless	1,605	672	–10.2	64.1	23.3	.260	.63
SKCS size	mm	1,605	672	2.3	3.3	2.8	.003	.56
SKCS weight	mg	1,605	672	23.8	56.2	39.2	.110	.58
Test weight	kg hl^–1^	1,605	672	53.1	65.9	61.9	.040	.70
Grain protein	%	1,605	672	6.0	16.1	10.8	.040	.19
Milling traits								
Break flour yield	%	1,605	672	27.2	56.6	48.0	.104	.62
Total flour yield	%	1,605	672	54.2	76.8	69.6	.070	.61
Milling score	unitless	1,564	650	64.6	99.2	85.5	.120	.55
Flour parameters								
Ash	%	1,564	650	0.2	0.5	0.4	.001	.48
Flour protein	%	1,605	672	5.3	13.3	9.0	.030	.18
Flour sodium dodecyl sulfate sedimentation	g ml^–1^	1,605	672	4.1	22.3	10.3	.061	.58
Water solvent retention capacity	%	1,604	672	42.7	72.6	54.1	.070	.56
Flour swelling volume	ml g^–1^	1,604	671	12.8	26.8	18.9	.044	.46
Baking parameter								
Cookie diameter	cm	1,507	629	7.4	10.0	9.2	.007	.53

^a^ Standard error.

^b^ *H* ^2^ broad‐sense heritability.

These end‐use quality traits were measured following the procedures from the American Association of Cereal Chemists International (AACC International, 2008) and Kiszonas et al. (2013). Grain characteristics including SKCS hardness, SKCS size, and SKCS weight were measured based on 200 kernels per sample using SKCS 4100 (Perten Instruments) (AACC Approved Method 55‐31.01). Test weight was measured as grain weight divided by volume according to the AACC Approved Method 55‐10.01. Grain protein was measured using near infrared reflectance (AACC Approved Method 39‐10.01), calibrated using Dumas nitrogen and a conversion factor of 5.7.

The samples were milled on a modified Quadrumat senior experimental milling system (Brabender) (Jeffers & Rubenthaler, 1977) and the milling traits flour yield, break flour yield, and milling score were measured. Flour yield was calculated as the percentage of total flour by weight (break and reduction rolls). Break flour yield was calculated as the percentage of flour recovered from the break rolls. Milling score was a function of both flour yield and ash content (Kiszonas et al., 2013). Ash and flour protein were measured following the AACC Approved Method 08‐01.01 and 39‐11.01, respectively. The SDS sedimentation test, which assesses the gluten strength of flour, was measured according to AACC Approved Method 56‐60.01. Water SRC, which measures water affinity of the flour macro‐polymers (starch, arabinoxylans, gluten, and gliadins), was performed as described in AACC Approved Method 56‐11.02. Flour swelling volume, which evaluates starch composition and hydrothermal swelling of flour samples, was performed as described in AACC Approved Method 56‐21.01. In soft wheat, the baking parameter, cookie diameter, is an essential indicator of soft wheat end‐use quality performance. Soft wheat flours with better overall quality will result in greater cookie diameter, which was measured according to AACC Approved Method 10‐52.02.

### 2.3 Genotyping

Genotyping was performed using the GBS protocol as described by Poland et al., 2021a). A subset of this germplasm (172 genotypes) was previously used for association mapping for SKCS hardness (Aoun et al., 2021). Thus, the genotyping of the plant material was as previously described by Aoun et al. (2021). The SNP marker physical positions were based on the *T. aestivum* RefSeq v1.0 reference genome (Appels et al., 2018). For further analysis, SNP data were filtered for missing data (≤30%), minor allele frequency (MAF ≥5%), and heterozygous frequency (≤15%). After marker filtering, 40,518 SNPs were left for further analysis (Supplemental Table S4). Missing data in the retained SNPs were imputed using the expectation–maximization algorithm (Poland et al., 2012b) implemented in the rrBLUP software package (Endelman, 2011) in R version 4.0.2 (R Core Team).

### 2.4 Phenotypic data analysis

#### 2.4.1 Mixed linear model

The data set based on all genotypes, environments, and traits was analyzed using mixed linear model (MLM) in ASReml‐R version 4 (Butler et al., 2018). The model was written as:

$$y=X\beta +Z_{\mathrm{genotype}}u+Z_{\mathrm{Env}}b+\varepsilon$$

where **y** represents the raw phenotypic observations for the trait and **β** includes a fixed effect for the intercept **X** (overall mean). The vector **u** and the corresponding matrix **Z_genotype_** represent the random effect for the genotypes, where $u∼N(0,{I\sigma }_{u}^{2})$, and **I** is the identity matrix. Environment (year–location combination) effects were incorporated in the model as random with the matrix **Z_Env_** and the effects vector $b∼N(0,{I\sigma }_{b}^{2})$. **Z_genotype_** and **Z_Env_** are incidence matrices relating the phenotype to the random effect **u** and **b** , respectively. The residual **ε** was distributed as $\varepsilon ∼N(0,{I\sigma }_{\varepsilon }^{2})$. The random effects in MLM are independent and identically distributed. The MLM was also used to extract the best linear unbiased predictions (BLUP) of the genotypes for each trait. Broad sense heritability (*H* ^2^) of each trait in this study were estimated using the method of Cullis et al. (2006) . The R code to estimate $H_{Cullis}^{2}$were as described by Schmidt et al. (2019) . The equation for heritability was:


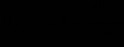

where ${\bar{v}}_{\Delta ..}^{\mathrm{BLUP}}$ is the mean‐variance of BLUP and 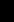
is genotypic variance (Cullis et al., 2006). The correlations were plotted using the ‘corrplot’ package (Wei et al., 2017) in R. Since the data was unbalanced, we used BLUP of the genotypes for the correlation analysis. Correlation values were considered significantly different from zero at *P* value ≤ .05.

#### 2.4.2 Factor analytic model

Factor analytic models (FA) are referred to as FA*k* where *k* is the number of latent factors. The FA models were used to calculate the variance–covariance matrix (**G_j_**) for the *j* environments using ASReml‐R. This matrix was then used to investigate the genetic correlation between environments and therefore the patterns of G × E (Beeck et al., 2010; Cullis et al., 2010; Heslot et al., 2013) for each of the end‐use quality traits in this dataset. For the FA model of order *k*, **G_j_** = **Λ**′ + **Ψ**, where **Λ** is a *j* × *k* matrix containing the environment loadings for the *k*th factor and **Ψ** is a diagonal matrix with different nonnegative parameters on the diagonal. In the FA models, genotypes were considered random, whereas environments were fitted as fixed effects. We assumed homogeneous error variances in the FA models and tested one to four factor analytic components. It was not possible to get the FA models to converge with heterogeneous error variances even for the one‐factor model. The FA models were compared to MLM based on Akaike information criterion (AIC), Bayesian information criterion (BIC), residual log‐likelihood, and percentage of variation explained by the models. Models with lower AIC, BIC, and residual log‐likelihood and higher percentage of variation explained were the best models. The best FA model with *k* number of factors was selected to estimate the genetic correlation between environment pairs for each end‐use quality trait. To test whether there is correlation between overlapping genotypes and genetic correlation estimates, a Mantel test (Mantel, 1967) was performed for each trait.

### 2.5 Environment clustering analysis

To investigate the relationship between environments for each end‐use quality trait, we performed environment clustering analysis to identify possible outlier environments or possible environment grouping. Genomic prediction was used to calculate genomic estimated breeding value (GEBV) of all genotypes in each environment based on genomic relationship matrix between genotypes as described by Dawson et al. (2013). This allowed the generation of complete distance matrix between environments that was later used for environment clustering analysis. The realized additive relationship matrix was calculated using ‘A.mat’ function as described by Endelman & Jannink (2012) in the R package rrBLUP (Endelman, 2011) based on 40,518 SNPs. For the genotypes that were evaluated in multiple nurseries in an environment, the genotype arithmetic mean across nurseries within a particular environment was used as the phenotypic data in the genomic BLUP model (GBLUP) using ‘mixed.solve’ function in rrBLUP package (Endelman, 2011). In this case our training and validation sets were fixed and therefore no iterations were used in the genomic prediction. The GEBV for every genotype in each environment were then calculated as described by Dawson et al. (2013) and were then used to compute a Euclidean distance matrix between environments. The Ward's clustering method was used on the distance matrix among environment with the function ‘hclust’ in R.

### 2.6 Genomic prediction of end‐use quality traits

For each quality trait we performed genomic prediction using GBLUP model with the function ‘emmreml’ in the R package EMMREML (Akdemir & Godfrey, 2015). The GBLUP model was as follows: y=Xβ+Zg+ε, where **y** is a vector of the raw phenotypic data, **β** is the vector of fixed effects for the environment with the incidence matrix **X**, the vector **g** is the random effect representing the GEBV for each genotype, **Z** is an incidence matrix connecting observations to genomic values, and **ε** is a vector of the residuals. The GEBV was calculated under the assumption $g∼(0,{K\sigma }_{g}^{2})$, where the additive genetic variance and $\sigma_{g}^{2}K$ is the square, symmetric genomic relationship matrix based on markers. The genomic relationship matrix was computed based on 40,518 SNPs using the function ‘A.mat’ function in the R package rrBLUP (Endelman, 2011).

To assess genomic prediction accuracy, we used 100 replicates of five‐fold cross‐validation. For each replicate, we randomly divided the population into five equal and mutually exclusive subsets or “folds.” The GBLUP model was then trained on four of the five subsets (training sets, 80% of the population size) to predict the fifth subset (validation set, 20% of the population size). For each replicate, the prediction accuracy was calculated as the Pearson's correlation coefficient between the GEBV predicted by the GBLUP model in the validation set and the corresponding estimated observed breeding values or BLUPs that were calculated using MLM. The prediction accuracy was averaged across the 100 replicates and was considered significantly different from zero at *P* value ≤ .05.

Preliminary results showed that for all traits except SKCS weight, ash, and flour swelling volume, the use of best linear unbiased estimators (BLUE) derived from FA model as the phenotype in GBLUP model did not improve genomic prediction accuracies compared to the use of BLUE derived from MLM. Therefore, we tested the effect of excluding outlier environments that were identified using (a) genetic correlations between environments and (b) clustering analysis based on Euclidian distance between environments on genomic prediction performance. Outlier environments were identified visually based on the heatmap of genetic correlations between environments (showing negative correlations with the remaining environments) and based on the dendrogram of the Euclidian distance between environments (distant environments from the remaining environments).

To further validate the outlier environments, we split the 29 environments into three subsets (closely related environments, outlier environments, and a randomly selected environment from the subset of closely related environments herein described as reference environment). Closely related environments were used as training to predict the outlier environments and the reference environment. Prediction accuracies of outliers were compared with that of the reference environment to conclude whether outlier exclusion was reasonable. We repeated this procedure for all traits in which outlier environments were detected. Genomic prediction in this case was as described above using GBLUP model in the R package EMMREML.

## 3 RESULTS

### 3.1 Phenotypic data analysis

Summary statistics for each of the end‐use quality traits are presented in Table 1. There was phenotypic variation in the germplasm for the 14 end‐use quality traits. Each of the traits followed a continuous distribution and the standard errors were relatively low. Broad‐sense heritability estimates ($H_{\mathrm{Cullis}}^{2}$) ranged from .18 to .70 (Table 1). The heritability was moderate to high for most traits and low for grain protein (.19) and flour protein (.18).

**FIGURE 1:**
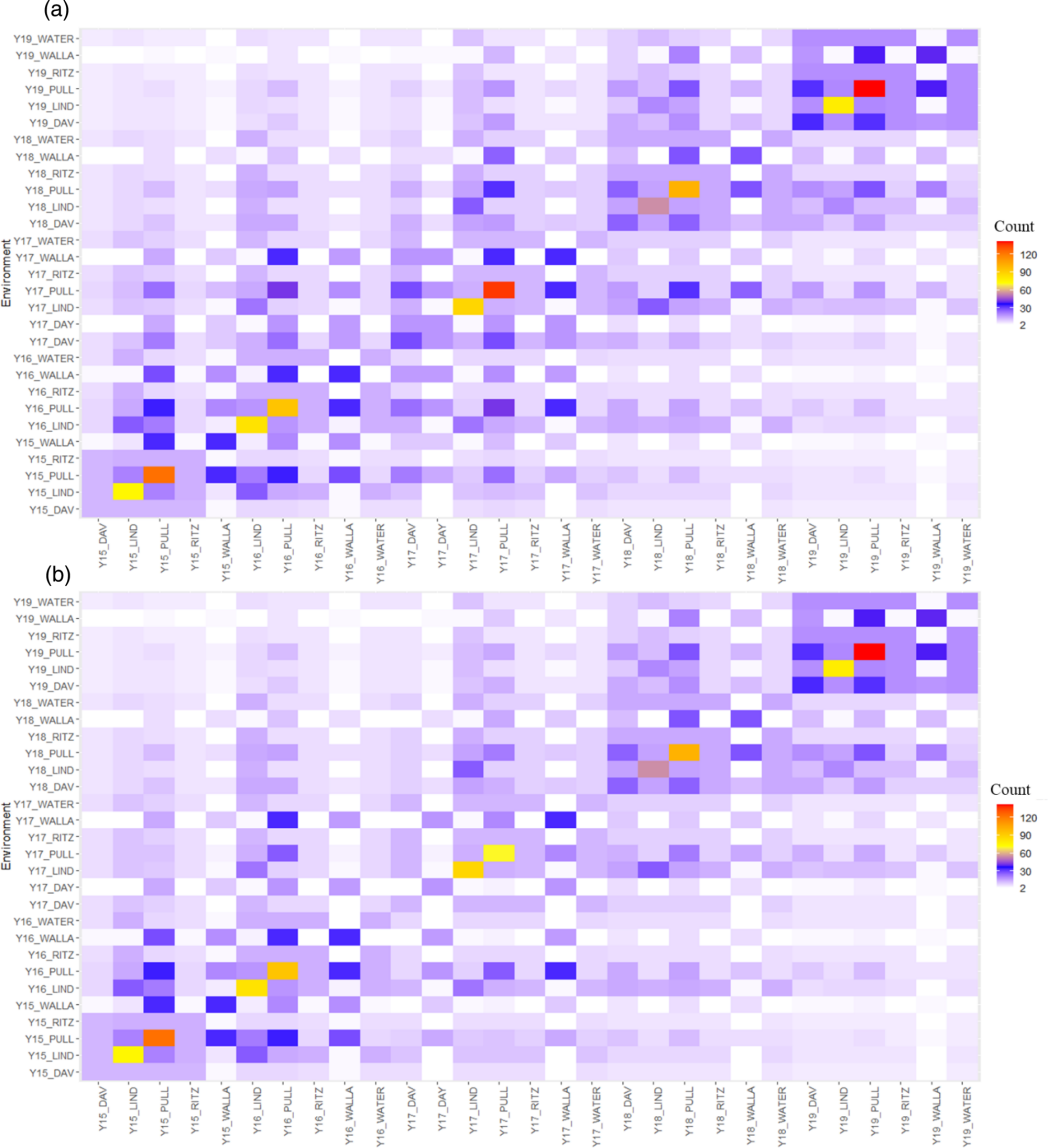
Connectivity of environments based on the number of shared genotypes. (a) Connectivity of environments for traits with the highest number of observations, i.e., test weight, Single Kernel Characterization System (SKCS) weight, grain protein, SKCS hardness, SKCS size, flour protein, flour sodium dodecyl sulfate sedimentation volume (SDS sedimentation), flour yield, and break flour yield. (b) Connectivity of environments for cookie diameter that had the least number of observations. White indicates the least connected environments and red indicates the most connected environments. Environments were written as year–location combination separated by underscore, i.e., the crop year 2015 (Y15), 2016 (Y16), 2017 (Y17), 2018 (Y18), and 2019 (Y19) were followed by the location Pullman (PULL), Lind (LIND), Davenport (DAV), Ritzville (RITZ), Waterville (WATER), Walla Walla (WALLA), and Dayton (DAY)

The connectivity between environments based on the overlap in genotypes grown is illustrated in the heatmap ranging from lowest in white to highest in red (Figure 1). There was generally good connectivity between the environments with a minimum of two shared genotypes and a maximum of 50 shared genotypes. The mean of shared genotypes between environments ranged from 10 to 13 lines depending on the trait (Figure 1; Supplemental Tables S2, S3).

**FIGURE 2:**
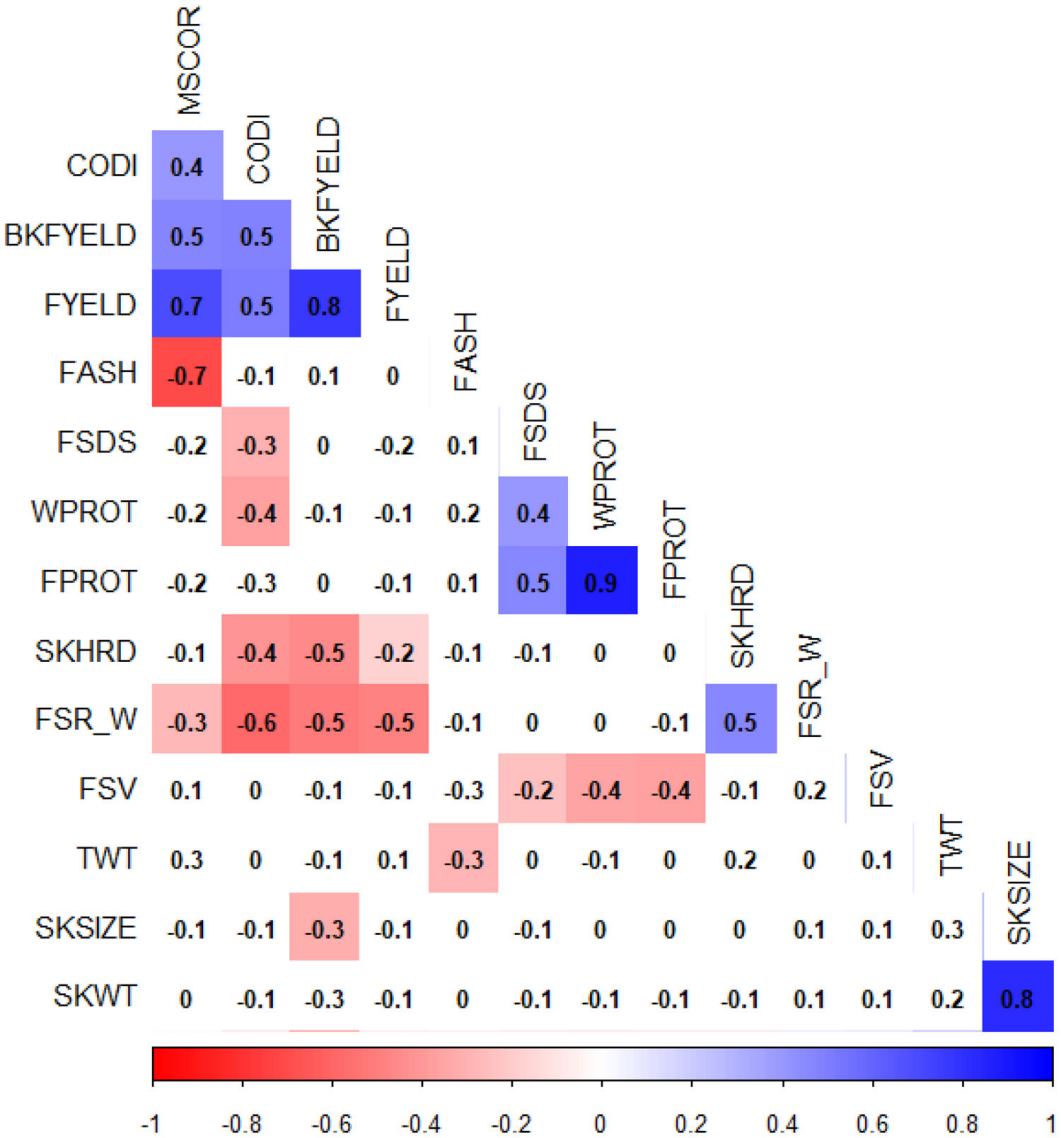
Phenotypic correlations between end‐use quality traits of soft white winter wheat genotypes based on best linear unbiased prediction across environments. The correlations in white color are not significant at the 95% confidence level (α = .05). The end‐use quality traits are Single Kernel Characterization System (SKCS) hardness (SKHRD), SKCS size (SKSIZE), SKCS weight (SKWT), test weight (TWT), grain protein (WPROT), break flour yield (BKFYELD), flour yield (FYELD), milling score (MSCOR), ash (FASH), flour protein (FPROT), flour sodium dodecyl sulfate sedimentation volume (FSDS), water solvent retention capacity (FSR_W), flour swelling volume (FSV), and cookie diameter (CODI)

Phenotypic correlations between the traits showed some significant negative and positive correlations that ranged from −.7 to −.2 and from .4 to .9, respectively (Figure 2). Significant positive correlations were observed between (a) flour yield, break flour yield, milling score, and cookie diameter, (b) SKCS hardness and water SRC, (c) SKCS weight and SKCS size, and (d) grain protein, flour protein, and SDS sedimentation. Significant negative correlations were found between milling score and ash. Similarly, cookie diameter was negatively correlated with SDS sedimentation, grain protein, SKCS hardness, and water SRC. We also found that break flour yield was negatively correlated with SKCS hardness, water SRC, and SKCS size. Flour yield was negatively correlated with SKCS hardness and water SRC, ash was negatively correlated with test weight, and FSV was negatively correlated with SDS sedimentation and grain and flour protein.

Model comparisons supported FA models over MLM (Supplemental Table S5). For most traits, the FA2 model was selected except for milling score, ash, and flour protein for which the FA3 was the best model, and cookie diameter for which FA4 was best (Table 2). The percent of variation accounted for by FA (84.6–99.4%) was higher compared with that for MLM (56–86%) (Table 2, Supplemental Table S5). The percent variation accounted for by FA*k* within each environment was calculated by each factor and across all considered *k* factors. The number of environments with less than 80% of variance explained by the factors in the models were only one to five environments out of the 29 environments that were listed in Table 2.

**TABLE 2:** Summary of the selected factor analytic (FA) models for each end‐use quality trait

Trait	Model	Residual log‐likelihood	BIC^a^	AIC^b^	% of variance explained	Environments with % of variation explained by the factors ≤80%^c^
SKCS hardness	FA2	–3,600.6	7,702.0	7,337.3	89.4	Y15_DAV and Y16_RITZ
SKCS size	FA2	2,843.6	–5,208.6	–5,557.2	84.6	Y15_WALLA and Y17_PULL
SKCS weight	FA2	–2,443.5	5,343.4	5,011.0	85.5	Y15_WALLA
Test weight	FA2	–821.9	2,144.4	1,779.7	89.9	Y15_PULL, Y17_PULL, Y19_DAV, and Y19_WATER
Grain protein	FA2	–502.2	1,497.6	1,138.4	89.2	Y15_DAV, Y17_PULL, Y18_WATER, Y19_RITZ, and Y19_WATER
Break flour yield	FA2	–1,838.1	4,162.1	3,808.1	94.0	Y15_PULL and Y16_PULL
Flour yield	FA2	–1,674.6	3,813.0	3,475.1	97.7	Y16_LIND and Y18_LIND
Milling score	FA3	–2,564.9	5,782.8	5,307.9	94.2	Y15_RITZ, Y16_RITZ, and Y16_WALLA
Ash	FA3	4,294.9	–7,929.5	–8,409.8	90.7	Y15_PULL and Y16_WATER
Flour protein	FA3	–265.5	1,208.4	715.0	88.1	Y17_RITZ and Y19_DAV
SDS sedimentation	FA2	–1,627.1	3,762.3	3,392.3	91.6	Y17_DAV, Y17_PULL, Y17_RITZ, Y19_PULL, and Y19_RITZ
Water SRC	FA2	–1,767.6	4,006.3	3,663.2	88.9	Y15_RITZ and Y17_DAY
FSV	FA2	–1,317.6	3,099.0	2,761.2	88.0	Y15_RITZ and Y17_PULL
Cookie diameter	FA4	1,705.9	–2,550.7	–3,175.9	99.4	Y18_LIND

^a^ Bayesian information criterion.

^b^ Akaike information criterion.

^c^ For the rest of the environments the percentage of variation accounted for by the factors in the model was >80%.

### 3.2 Genetic correlation between environments

**FIGURE 3:**
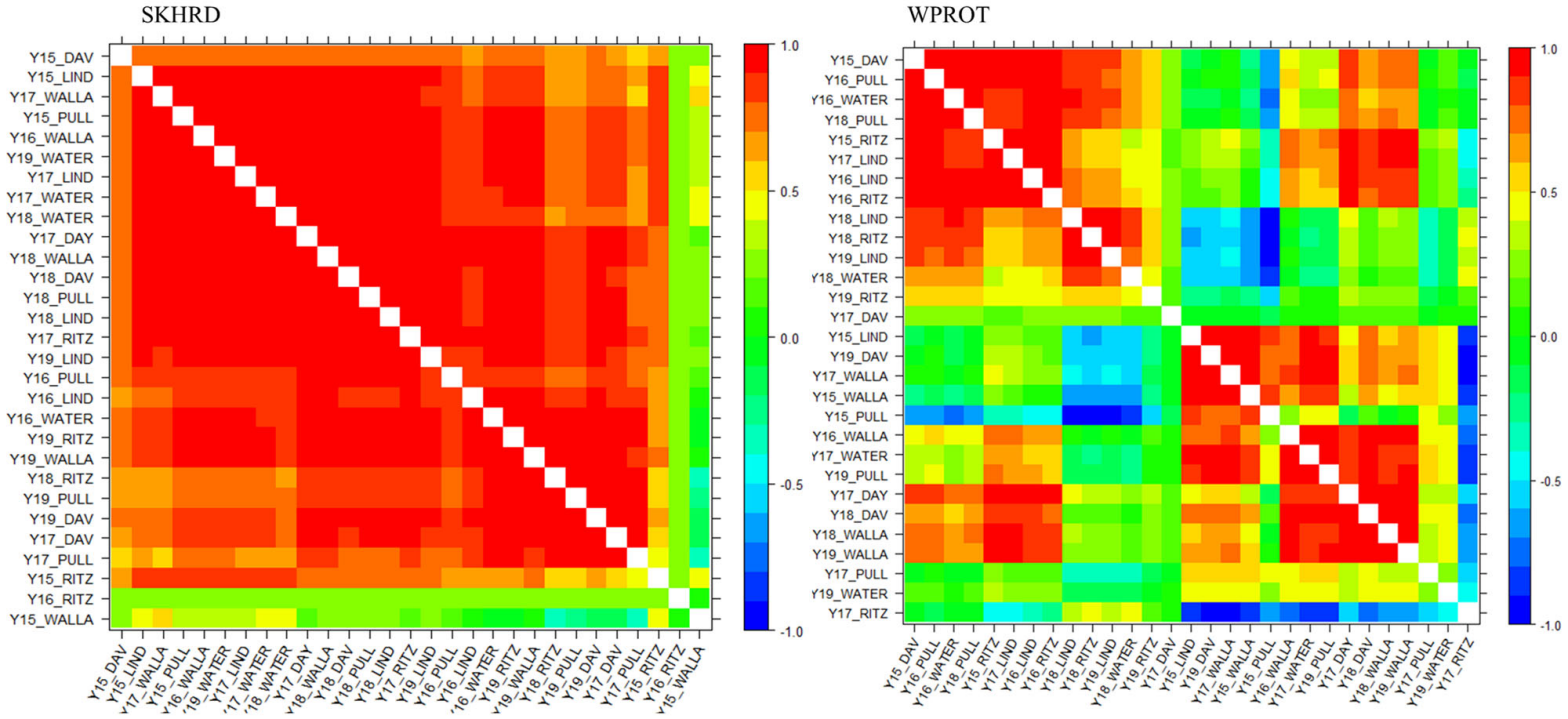
Heat map of genetic correlations between environments for SKCS hardness (SKHRD) and grain protein (WPROT). Key on the right‐hand side depicts the correlation color scale. Environments were written as year–location combination separated by underscore, i.e., the crop year 2015 (Y15), 2016 (Y16), 2017 (Y17), 2018 (Y18), and 2019 (Y19) were followed by the location Pullman (PULL), Lind (LIND), Davenport (DAV), Ritzville (RITZ), Waterville (WATER), Walla Walla (WALLA), and Dayton (DAY)

Genetic correlation between environments based on factor analytic models was used in this study to understand G × E interaction for the 14 end‐use quality traits. For all traits, Mantel test failed to reject the null hypothesis that there is no correlation between overlapping genotypes and genetic correlation estimates at the 95% confidence level (Supplemental Table S6). The pattern of heterogeneity of correlation between environments varied depending on the trait (Figure 3 and Supplemental Figure S1). For instance, there were strong positive correlations between most environments for SKCS hardness, test weight, break flour yield, SDS sedimentation, and water SRC, suggesting low G × E interaction. This was also reflected in high heritability estimates of these traits (.56–.70).

For SKCS size, SKCS weight, flour yield, and FSV, there were moderate levels of heterogeneity in the genetic correlation between environments and only a few environments were negatively correlated (blue color in the correlation heatmap in Figure 3 and Supplemental Figure S1). For instance, for SKCS size, the environment 2015 Ritzville (Y15_RITZ) and 2015 Lind (Y15_LIND) showed cross‐over G × E interaction (change in genotype rank) with environments Y16_RITZ, Y17_DAV, and Y17_PULL. For SKCS weight, Y15_RITZ showed a cross‐over G × E interaction with a few environments, mainly Y16_RITZ and Y17_PULL. This G × E interaction pattern for SKCS weight was highly similar to that observed for SKCS size. For flour yield, cross‐over G × E interaction was observed between Y15_RITZ and the three environments Y19_LIND, Y19_PULL, and Y19_WALLA, between Y15_WALLA and the two environments Y19_PULL and Y19_WALLA, and between Y15_PULL and Y19_WALLA. For FSV, cross‐over G × E interaction was observed between Y17_LIND and environments Y15_RITZ and Y18_LIND, and between Y15_RITZ and the two environments Y17_DAV and Y17_WATER.

For ash, milling score, and cookie diameter, there were relatively higher levels of heterogeneity of correlation between environments compared with the traits discussed above. For ash, the environment Y17_Lind showed a negative correlation with most of the other environments. For milling score, the four environments Y18_RITZ, Y17_LIND, Y18_WATER, and Y17_WATER were negatively correlated with most of the other environments. For cookie diameter, negative correlations were observed between Y18_WATER and environments Y15_DAV, Y15_PULL, Y15_WALLA, and Y17_WATER and between Y15_RITZ and Y17_DAV. The highest levels of heterogeneity between environments in this study were observed for grain protein and flour protein. This was also reflected in the heritability estimates that were the lowest (.18–.19) in this study.

### 3.3 Environment clustering analysis

**FIGURE 4:**
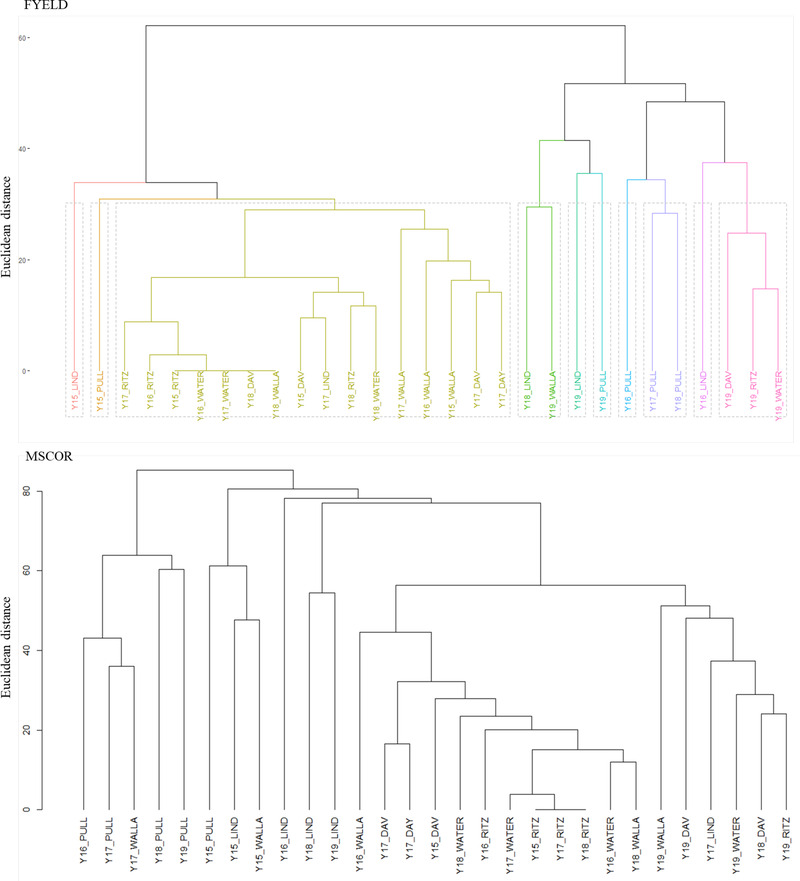
Environment clustering analysis for flour yield (FYELD) and milling score (MSCOR). Different environment clusters were illustrated with different colors. Environments were written as year–location combination separated by underscore, i.e., the crop year 2015 (Y15), 2016 (Y16), 2017 (Y17), 2018 (Y18), and 2019 (Y19) were followed by the location Pullman (PULL), Lind (LIND), Davenport (DAV), Ritzville (RITZ), Waterville (WATER), Walla Walla (WALLA), and Dayton (DAY)

Based on Ward's clustering method, no significant environment clustering was observed for any trait except flour yield (Figure 4 and Supplemental Figure S2). However, for some traits we could identify some outlier environments that were distant from the remaining environments based on Euclidian distance. Environment clustering analysis showed no obvious outlier environments for SKCS hardness, test weight, break flour yield, SDS sedimentation, and water SRC, which agreed with the results from the genetic correlation between environments. In contrast to the results from the genetic correlation between environments, there were no outlier environments based on environment clustering for milling score and cookie diameter. Environment clustering analysis showed outlier environments for the remaining traits. For SKCS size, SKCS weight, grain protein, ash, flour protein, FSV, and flour yield, the identified outlier environments based on clustering analysis were also considered outliers based on genetic correlations between environments (Table 3).

**TABLE 3:** Effect of excluding outlier environments on genomic prediction accuracies of end‐use quality traits

	No. of genotypes^a^			Excluded environments^b^		Genomic prediction accuracies^c^		
Trait	Original	Environment genetic correlation	Environment clustering analysis	Environment genetic correlation	Environment clustering analysis	Original	Environment genetic correlation	Environment clustering analysis
SKCS hardness	672	672	672	–	–	.58	–	–
SKCS size	672	635	672	Y15_WALLA, Y15_RITZ, Y15_LIND	Y15_WALLA	.55	.59	.57
SKCS weight	672	672	672	Y15_WALLA, Y15_RITZ	Y15_WALLA	.56	.57	.57
Test weight	672	672	672	–	–	.57	–	–
Grain protein	672	564	618	Y15_PULL, Y15_WALLA, Y17_WALLA, Y19_DAV, Y15_LIND, Y17_RITZ	Y15_PULL	.41	.40	.37
Break flour yield	672	672	672	–	–	.57	–	–
Flour yield	672	606	578	Y15_PULL, Y15_RITZ, Y15_WALLA	Y15_LIND, Y15_PULL	.59	.59	.58
Milling score	650	598	650	Y17_LIND, Y18_WATER, Y18_RITZ, Y17_WATER	–	.48	.52	–
Ash	650	598	598	Y17_LIND	Y17_LIND	.39	.50	.50
Flour protein	672	560	618	Y15_PULL, Y18_PULL, Y15_Walla	Y15_PULL	.37	.37	.34
SDS sedimentation	672	672	672	–	–	.58	–	–
Water SRC	672	672	672	–	–	.48	–	–
FSV	671	619	619	Y17_LIND, Y15RITZ	Y17_LIND	.49	.52	.52
Cookie diameter	629	629	629	Y18_WATER	–	.52	.52	–

^a^ Number of genotypes in the original dataset and after excluding outlier environments based on genetic correlation between environments (environments with negative correlations with the remaining environments illustrated in blue color in the genetic correlation heatmap in Figure 3; Supplemental Figure S1) and environment clustering analysis (distant environments from the remaining environments in Figure 4; Supplemental Figure S2).

^b^ Excluded outlier environments based on genetic correlations between environments and environment clustering analysis. For some traits none of the environments were excluded (no outlier environments were denoted with ‘–‘).

^c^ Genomic prediction accuracies in the original dataset and after excluding outlier environments based on genetic correlation between environments and environment clustering analysis.

### 3.4 Genomic prediction for end‐use quality traits

#### 3.4.1 Genomic prediction based on the whole dataset

**FIGURE 5:**
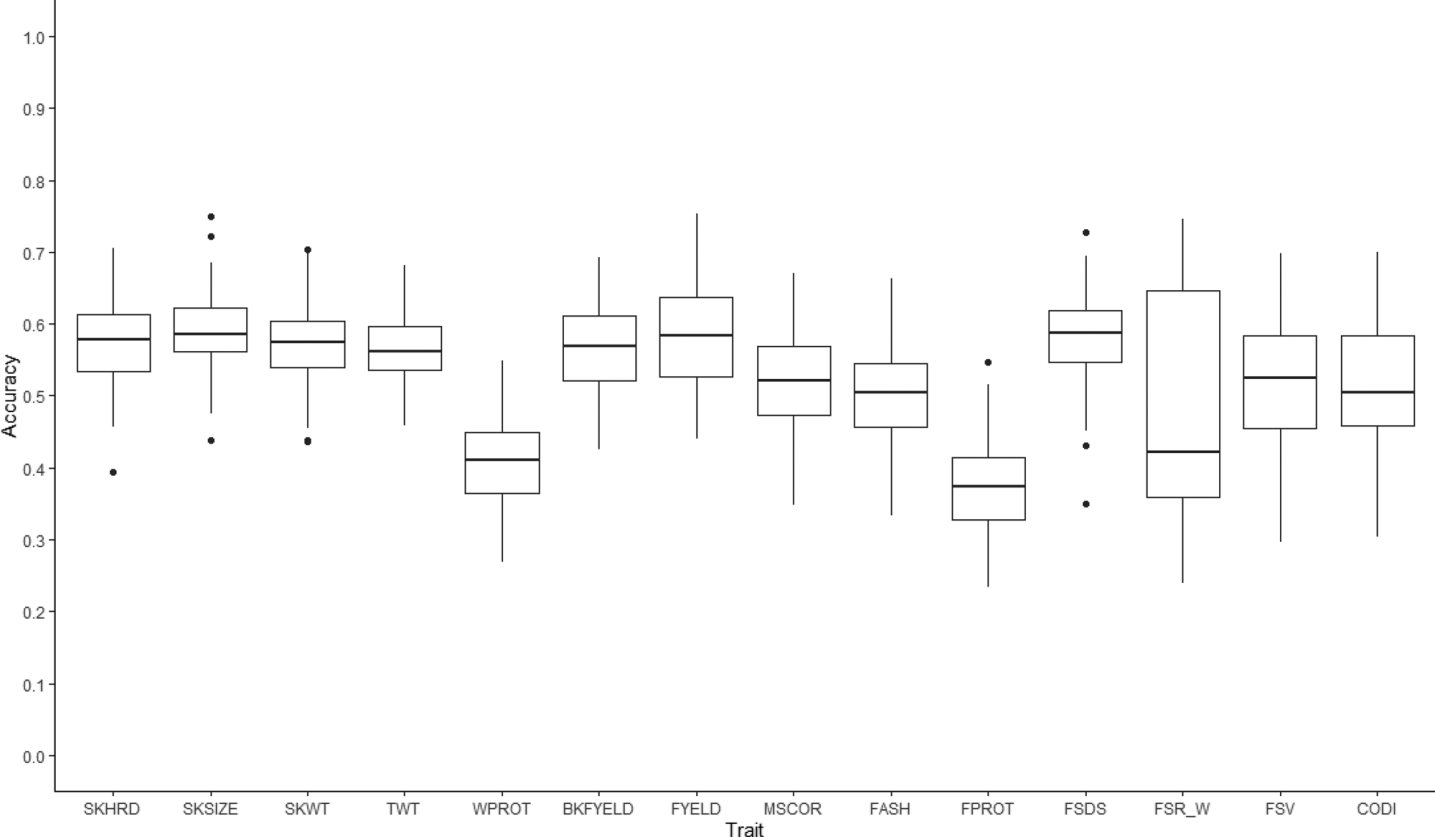
Genomic prediction accuracies for end‐use quality traits. Single Kernel Characterization System (SKCS) hardness (SKHRD), SKCS size (SKSIZE), SKCS weight (SKWT), test weight (TWT), grain protein (WPROT), break flour yield (BKFYELD), flour yield (FYELD), milling score (MSCOR), ash (FASH), flour protein (FPROT), flour sodium dodecyl sulfate sedimentation volume (FSDS), water solvent retention capacity (FSR_W), flour swelling volume (FSV), and cookie diameter (CODI)

Genomic prediction accuracies of the end‐use quality traits using GBLUP model were all significant and ranged from .37 to .59 depending on the trait (Figure 5 ). The highest prediction accuracies (*r *= .52–.59) were found for SKCS hardness, SKCS size, SKCS weight, test weight, break flour yield, flour yield, SDS sedimentation, and cookie diameter. For grain protein, milling score, ash, water SRC, and FSV, the accuracies ranged from .39 to .49. The lowest genomic prediction accuracy (.37) was for flour protein.

**FIGURE 6:**
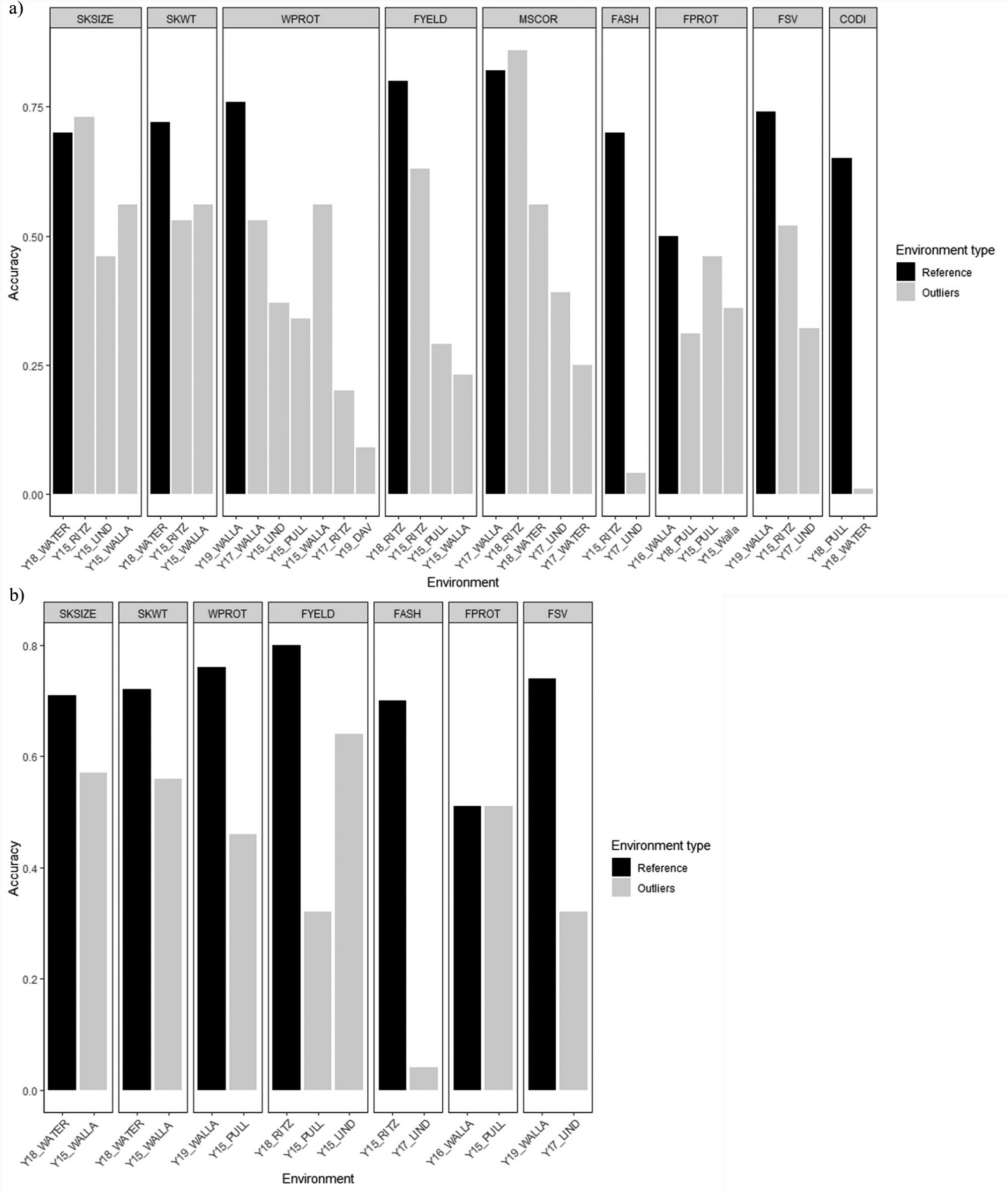
Genomic prediction accuracies of outlier environments based on the remaining closely related environments for nine end‐use quality traits. (a) Outlier environments were detected based on genetic correlations between environments. (b) Outlier environments were detected based on clustering analysis. Reference environment was randomly selected from the subset of closely related environments and was used as the baseline for prediction accuracy comparison. This analysis was performed for Single Kernel Characterization System (SKCS) size (SKSIZE), SKCS weight (SKWT), grain protein (WPROT), flour yield (FYELD), milling score (MSCOR), ash (FASH), flour protein (FPROT), flour swelling volume (FSV), and cookie diameter (CODI) in which outlier environments as described Table 3

#### 3.4.2 Effect of excluding outlier environments on genomic prediction performance

Outlier environments identified using genetic correlation between environments and environment clustering analysis are listed in Table 3. Although excluding outlier environments resulted in a reduction in the number of genotypes used to train the GBLUP model, it improved in many cases the genomic prediction accuracies. Excluding outlier environments based on genetic correlation between environments provided higher and more consistent increases in genomic prediction accuracies compared with that based on environment clustering analysis (Table 3).

Excluding outlier environments based on genetic correlations between environments resulted in many cases in an increase in genomic prediction accuracies by 1–11%. The increase in prediction accuracies for SKCS size, SKCS weight, milling score, ash, and FSV was 4, 1, 4, 11, and 3%, respectively. The prediction performance did not improve for grain protein, flour yield, flour protein, and cookie diameter by excluding outlier environments. The lack of improvement in genomic prediction accuracies for the latter four traits was most likely due to the remaining moderate heterogeneity between the non‐excluded environments as illustrated in Figure 3 and Supplemental Figure S1.

Excluding outlier environments based on the environment clustering approach resulted in increased genomic prediction accuracies for SKCS size, SKCS weight, ash, and FSV by 2, 1, 11, and 3%, respectively (Table 3). For grain protein, flour yield, and flour protein excluding outlier environments resulted in 4, 1, and 3% reduction in the prediction accuracies. As discussed above, extreme environment clustering was observed for flour yield (Figure 4). Consequently, excluding only two outlier environments (Y15_LIND and Y15_PULL) was not sufficient to account for environment heterogeneity. Similarly, the failure to increase genomic prediction for grain protein and flour protein by excluding outlier environments is likely associated with high G × E interaction (Figure 3, Supplemental Figure S1) that could not be overcome by excluding a few outlier environments.

Across all traits in which outlier environments were identified, prediction accuracies of outliers based on the remaining environments were lower compared with the reference environment (Figure 6). The only exception was for the outliers Y15_RITZ and Y18_RITZ for SKCS size and milling score, respectively, in which prediction accuracies were slightly better compared with reference environments. This analysis further validated the outlier environments identified using both approaches, i.e., genetic correlation and clustering analysis.

## 4 DISCUSSION

Extracting valuable information from unbalanced and multi‐environment trial datasets is challenging in breeding programs (Sjoberg et al., 2021). In this study we examined the pattern of G × E interaction in soft white wheat end‐use quality traits using methods that can accommodate unbalanced and multi‐environment data. We found that the FA model was an effective tool to examine G × E interactions by studying the genetic correlation between environments. The FA model accounted for higher variation in the data (84.6–99.4%) compared with MLM (56–86%). This agrees with observations by Sjoberg et al. (2021), which showed that FA model explained more variation in the model compared with MLM for wheat falling number. The advantages of FA models in comparison with MLM were discussed by Meyer (2009), who argued that FA models outperformed MLM in modeling G × E in all types of datasets, including those with missing data or with multiple random effects. Similarly, Kelly et al. (2007) applied FA models to multi‐environment trial datasets in multiple crops and found it to be more effective in handling G × E interactions compared with other models. Although not tested in this study, FA models were also shown to be useful in studying genotype overall performance and stability across environments, thus enabling more effective selection in a breeding program (Burgueño et al., 2008; Dias et al., 2018; Mengesha et al., 2019; Sjoberg et al., 2021; Smith & Cullis, 2018).

Based on genetic correlations between environments, G × E interaction was negligible for the end‐use quality traits SKCS hardness, test weight, break flour yield, SDS sedimentation, and water SRC. This implies that selection for these traits can rely on testing in fewer environments. For the remaining traits, especially grain protein and flour protein, where high G × E interaction was observed, selection should be based on observations in multiple environments. Negative correlations between some environment pairs are the most problematic form of G × E interaction illustrating change in genotype rank across environments, which makes selection much more challenging for these traits. Our study also showed that genomic prediction of an environment is higher when model training was performed based on data from closely related environments. Therefore, understanding the pattern of G × E interaction is helpful to optimize genomic prediction performance. The heterogeneity between environments for some end‐use quality traits could be explained by differences in climate (temperature and precipitation), soil characteristics, and disease pressure during the growing season. For instance, in traits where G × E interaction was high, many of the identified outlier environments were in crop year 2015 (e.g., Y15_PULL, Y15_Lind, Y15_Walla, and Y15_RITZ). This could be associated with the low precipitation in 2015, which was the lowest in the month of May since 2005 (https://www.enlight‐inc.com/total_rainfall_2007.html). Disease pressure could be another factor that contributed to the variation between environments. For example, snow mold is known to be prevalent in the northern part of WA such as in Waterville where snow mold of wheat has been a chronic occurrence since the 1940s. Stripe rust, another major wheat disease in the Pacific Northwest of the United States, is affected by weather conditions (Chen et al., 2014). Stripe rust severity is higher in locations with moist weather conditions such as Pullman and lower in locations with dry weather conditions such as in Lind. Although diverse factors can explain the variability between environments in this study, their direct effects on end‐use quality traits are not well understood.

Genomic prediction in this study has sufficient accuracies to justify implementing genomic selection in breeding for end‐use quality traits in soft white wheat. Genomic selection can be implemented to enable more effective selection for end‐use quality in earlier generations and in a broader array of genotypes. Thus, genomic selection has the potential to enhance the rate of genetic gains for end‐use quality traits. Excluding outlier environments based on genetic correlation was more advantageous in improving genomic prediction accuracies compared with excluding outlier environments based on environment clustering analysis. This could be due to the small training population sizes in some of the environments (i.e., Ritzville and Waterville locations) that affected within environment prediction accuracies. Shengqiang et al. (2009) reported that there is a positive correlation between training population size and genomic prediction accuracy. However, we found that most of the detected outlier environments using environment clustering analysis were also identified as outliers using genetic correlation approach. This suggests that even with the challenge of small population size in some of the environments, genomic prediction approach was successful in characterizing environments. The high relatedness between lines in this breeding program could compensate for small population sizes within environments.

Dawson et al. (2013) found that accounting for G × E interaction did not increase genomic prediction accuracies for yield. However, Heslot et al. (2013) found that excluding outlier environments resulted in higher prediction accuracy of wheat yield. In this study we found that the gain in prediction accuracies depended on the trait. For instance, accounting for G × E interaction by excluding outlier environments improved genomic prediction by 1–11% for SKCS size, SKCS weight, milling score, ash, and FSV that were less affected by environment. However, for other traits (grain and flour protein, flour yield, and cookie diameter) there was no advantage in excluding outlier environments on the prediction accuracies. Both approaches used to characterize environments in this study could be used to identify mega‐environments in breeding programs that test in several distinct environments. Subsequently, separate genomic prediction models can be performed within defined mega‐environments as proposed by Lado et al. (2016). Other methods to incorporate G × E interaction in genomic prediction models have been suggested. Burgueño et al. (2012) proposed a single‐step mixed model for computing GEBV while modeling the G × E by a factor analytic model and have reported that this model increased prediction accuracies. However, Heslot et al. (2013) argued that fitting such a complex model for large and unbalanced data sets was not appropriate. Other studies modeled G × E interaction in genomic selection models by including environmental factors (e.g., location latitude and longitude and weather data) as cofactors in the models (Heslot et al., 2014; Jarquin et al., 2014). Reproducing Kernel Hilbert Spaces (RKHS) with Gaussian kernels that incorporate G × E interaction were reported to provide better prediction performance compared with linear GBLUP (Cuevas et al., 2016, 2020). Testing diverse methods to accommodate G × E interaction in genomic selection models for end‐use quality traits that are highly affected by the environment is warranted in future studies.

## 5 CONCLUSION

We examined the pattern of G × E interaction for 14 end‐use quality traits in a soft white winter wheat breeding program. Genetic correlations between environments based on FA models showed low to moderate G × E interaction for most traits but high G × E interaction for other traits, especially grain and flour protein. Outlier environments were identified using genetic correlation between environment and clustering analysis. The genomic prediction accuracies achieved in this study justify the application of genomic selection for soft white wheat end‐use quality traits. Excluding outlier environments based on genetic correlation between environments was much more effective in increasing genomic prediction accuracies compared with environment clustering analysis. Other methods to accommodate G × E interaction in genomic prediction models could be tested in future studies.

## AUTHOR CONTRIBUTIONS

Meriem Aoun: Conceptualization; Formal analysis; Methodology; Writing‐original draft; Writing‐review & editing. Arron Carter: Resources; Writing‐review & editing. Yvonne A. Thompson: Formal analysis; Writing‐review & editing. Brian Ward: Data curation; Writing‐review & editing. Craig F. Morris: Conceptualization; Methodology; Resources; Writing‐review & editing.

## CONFLICT OF INTEREST

The authors declare that there is no conflict of interest.

## Supporting information


**Supplemental Figure S1**. Heat map of genetic correlations between environments for end‐use quality traits.
**Supplemental Figure S2**. Environment clustering analysis for end‐use quality traits.
**Supplemental Table S1**. Phenotypic data for 14 end‐use quality traits in soft white winter wheat.
**Supplemental Table S2**. Connectivity of environments for traits with the highest number of observations.
**Supplemental Table S3**. Connectivity of environments for cookie diameter that had the least number of observations.
**Supplemental Table S4**. Genotypic data of 672 soft white winter wheat genotypes.
**Supplemental Table S5**. Summary of fitted mixed linear model and factor analytic models.
**Supplemental Table S6**. Mantel test for the correlations between overlapping genotypes between environments and genetic correlation estimates.

## Data Availability

Data are available in supplemental material.
