# Supplementary material for: Environment characterization and genomic prediction for end‐use quality traits in soft white winter wheat
Source: Plant Genome. 2021 Aug 15;14(3):e20128. doi: 10.1002/tpg2.20128 (PMC12806971; doi:10.1002/tpg2.20128)
Supplement: Supplementary file 1 [file TPG2-14-e20128-s001.zip › tpg220128-sup-0001-FigureS1-S2.docx]

**Supplementary** **Figure S1****.** Heat map of genetic correlations between environments for end-use quality traits. Key on the right-hand side depicts the correlation color scale. The end-use quality traits are Single Kernel Characterization System (SKCS) size (SKSIZE), SKCS weight (SKWT), test weight (TWT), break flour yield (BKFYELD), flour yield (FYELD), milling score (MSCOR), ash (FASH), flour protein (FPROT), flour sodium dodecyl sulfate sedimentation volume (FSDS), water solvent retention capacity (FSR_W), flour swelling volume (FSV), and cookie diameter (CODI). Environments were written as year-location combination separated by underscore, ie., the crop year 2015 (Y15), 2016 (Y16), 2017 (Y17), 2018 (Y18), and 2019 (Y19) was followed by the location Pullman (PULL), Lind (LIND), Davenport (DAV), Ritzville (RITZ), Waterville (WATER), Walla Walla (WALLA), and Dayton (DAY).

**Supplementary Figure S2.** Environment clustering analysis for end-use quality traits. The end-use quality traits are Single Kernel Characterization System (SKCS) hardness (SKHRD), SKCS size (SKSIZE), SKCS weight (SKWT), test weight (TWT), grain protein (WPROT), break flour yield (BKFYELD), ash (FASH), flour protein (FPROT), flour sodium dodecyl sulfate sedimentation volume (FSDS), water solvent retention capacity (FSR_W), flour swelling volume (FSV), and cookie diameter (CODI). Environments were written as year-location combination separated by underscore, ie., the crop year 2015 (Y15), 2016 (Y16), 2017 (Y17), 2018 (Y18), and 2019 (Y19) was followed by the location Pullman (PULL), Lind (LIND), Davenport (DAV), Ritzville (RITZ), Waterville (WATER), Walla Walla (WALLA), and Dayton (DAY).
